# *Leishmania (Viannia) naiffi*: rare enough to be neglected?

**DOI:** 10.1590/0074-02760150128

**Published:** 2015-09

**Authors:** Giselle Aparecida Fagundes-Silva, Gustavo Adolfo Sierra Romero, Elisa Cupolillo, Ellen Priscila Gadelha Yamashita, Adriano Gomes-Silva, Jorge Augusto de Oliveira Guerra, Alda Maria Da-Cruz

**Affiliations:** 1 Fundação Oswaldo Cruz, Instituto Oswaldo Cruz, Laboratório Interdisciplinar de Pesquisas Médicas, Rio de Janeiro, RJ, Brasil; 2 Universidade de Brasília, Faculdade de Medicina, Núcleo de Medicina Tropical, Brasília, DF, Brasil; 3 Fundação Oswaldo Cruz, Instituto Oswaldo Cruz, Laboratório de Pesquisas em Leishmaniose, Rio de Janeiro, RJ, Brasil; 4 Fundação de Medicina Tropical Dr Heitor Vieira Dourado, Gerência de Leishmaniose, Manaus, AM, Brasil; 5 Fundação Oswaldo Cruz, Instituto Nacional de Infectologia Evandro Chagas, Plataforma de Laboratório Multiusuário, Rio de Janeiro, RJ, Brasil

**Keywords:** Leishmania (Viannia) naiffi, therapeutic failure, clinical outcome, multilocus enzyme electrophoresis, Amazon Region

## Abstract

In the Brazilian Amazon, American tegumentary leishmaniasis (ATL) is endemic and presents a wide spectrum of clinical manifestations due, in part, to the circulation of at least seven* Leishmania *species. Few reports of *Leishmania (Viannia) naiffi *infection suggest that its occurrence is uncommon and the reported cases present a benign clinical course and a good response to treatment. This study aimed to strengthen the clinical and epidemiological importance of *L. (V.) naiffi *in the Amazon Region (Manaus, state of Amazonas) and to report therapeutic failure in patients infected with this species. Thirty *Leishmania* spp samples isolated from cutaneous lesions were characterised by multilocus enzyme electrophoresis. As expected, the most common species was *Leishmania (V.) guyanensis* (20 cases). However, a relevant number of*L. (V.) naiffi* patients (8 cases) was observed, thus demonstrating that this species is not uncommon in the region. No patient infected with*L. (V.) naiffi *evolved to spontaneous cure until the start of treatment, which indicated that this species may not have a self-limiting nature. In addition, two of the patients experienced a poor response to antimonial or pentamidine therapy. Thus, either ATL cases due to *L. (V.) naiffi *cannot be as uncommon as previously thought or this species is currently expanding in this region.

American tegumentary leishmaniasis (ATL) is highly endemic in the state of Amazonas (AM), Brazil. According to the Information System on Notifiable Diseases database, 18,675 Brazilian cases were reported in 2013, 8% of which occurred in the city of Manaus, AM (SVS/MS 2015). In this region, control of the disease is considered difficult because of the epidemiological characteristics and socioeconomic conditions associated with the sylvatic transmission cycle. In addition, environmental changes may influence infection dynamics, which makes control even more difficult. The circulation of at least seven *Leishmania *species has been observed in ATL in the Amazon Region. *Leishmania (Viannia) guyanensis*is the most prevalent species and is highly endemic in north of the Amazon River (Naiff et al. 1988, Grimaldi Jr et al. 1991, Lainson et al. 1994, Romero et al. 2001a, 2002a,Guerra et al. 2003). In this sense, several studies have shown that the frequency of circulating species in this area varies (Table I) and that *Leishmania (V.) naiffi* seems to be more consistently isolated from cutaneous lesions in patients from this region.

**TABLE I:** Frequency of *Leishmania *species associated with American tegumentary leishmaniasis in Amazon Region

*Leishmania*species	Cases (n)	Relative proportion within the study (%)	Region of endemicity state (city)	Reference
*L. (Viannia) guyanensis*	61	64.9	Amapá, Amazonas, Pará and Rondônia	Grimaldi Jr et al. (1991)
*L. (V.) braziliensis*	15	16		
*L. (V.) naiffi*	10	10.6		
*L. (Leishmania) amazonensis*	6	6.4		
Not identified	2	2.1		
*L. (V.) guyanensis*	69	97.2	Amazonas (Manaus)	Romero et al. (2002b)
*L. (V.) braziliensis*	2	2.8		
*L. (V.) braziliensis*	16	53.3	Acre (Rio Branco)	da Silva et al. (2006)
*L. (V.) lainsoni*	12	40		
*L. (V.) guyanensis*	1	3.3		
Hybrid:* L. (V.) lainsoni/L. (V.) naiffi*	1	3.4		
*L. (V.) guyanensis*	10	76.9	Amazonas (Manaus)	Figueira et al. (2008)
*L. (V.) naiffi*	3	23.1		
*L. (V.) guyanensis*	8	80	Amazonas (Rio Preto da Eva)	Figueira et al. (2008)
*L. (V.) naiffi*	2	20		
*L. (V.) guyanensis*	80	89	Amazonas (Rio Preto da Eva)	Figueira et al. (2014)
*L. (L.) amazonensis*	7	7.8		
*L. (V.) naiffi*	3	3.3		
*L. (V.) braziliensis*	11	25.6	Pará (lower Amazon Region)	Jennings et al. (2014)
*L. (V.) guyanensis*	6	13.9		
*L. (V.) shawi*	4	9.3		
*L. (V.) shawi*	7	16.3		
*L. (V.) lainsoni*	6	13.9		
*L. (L.) amazonensis*	2	4.7		
Hybrid:* L. (V.) guyanensis/ L. (V.) shawi shawi*	7	16.3		

*L. (V.) naiffi *was first described by Lainson and Shaw (1989) after being isolated from an armadillo (*Dasypus novemcinctus*) in the state of Pará, northern Brazil. The few cases described in the literature usually associate *L. (V.) naiffi*with low rates of virulence in humans*. *Thus, it has been described that the disease evolves with a benign clinical course and a good response to treatment (Naiff et al. 1991, Pratlong et al. 2002). Furthermore, two cases of spontaneous healing of leishmaniasis caused by *L*.* (V.) naiffi* were described (van der Snoek et al. 2009). To date, however, no association between *L. (V.) naiffi* and mucosal leishmaniasis has been observed. *L. (V.) naiffi* cutaneous leishmaniasis lesions are usually ulcerated, unique, small and located on the hands, arms or legs (Naiff et al. 1991). In the same way, experimental studies have shown that *L. (V.) naiffi* frequently causes discrete or even nonapparent infections on hamsters’ skin (Lainson & Shaw 1989). These findings were supported by in vitro analysis, which demonstrated that *L. (V.) naiffi* showed the lowest infection index and the highest nitric oxide production compared with other species of the *Viannia* subgenus (Campos et al. 2008). Then, the clinical and experimental information previously reported supported the idea that infection by *L. (V.) naiffi* commonly results in benign manifestations. Therefore, the present study aimed to strengthen the awareness of the clinical and epidemiological importance of *L. (V.) naiffi* in the Amazon Region and to report therapeutic failure associated with this species.

Thirty *Leishmania* spp samples were isolated from cutaneous lesions of patients from different surrounding areas of Manaus. Samples were collected during 2011-2013 at the Heitor Vieira Dourado Tropical Medicine Foundation (FMT-HVD), a reference centre for tropical diseases in AM. Skin lesion fragments obtained by biopsy were cultured in Novy-Neal-Nicolle medium and *Leishmania* was isolated. Parasites were sent to the *Leishmania* Collection from the Oswaldo Cruz Institute for species identification. This study was approved by the Research Ethical Committee of FMT-HVD (protocol 21273572). Identification of *Leishmania*spp isolates was based on multilocus enzyme electrophoresis (MLEE) and performed on agarose gel and allelic variations were tested for the following enzymes: 6-phosphogluconate dehydrogenase (EC1.1.1.44), glucose-6-phosphate dehydrogenase (E.C.1.1.1.49) and isocitrate dehydrogenase (E.C.1.1.1.42). The method was performed in accordance with the conditions described by Cupolillo et al. (1994).

Four species were identified: *L. (V.) guyanensis* 66.7% (20/30),*L. (V.) naiffi* 26.7% (8/30), *Leishmania (Leishmania) amazonensis* 3.3% (1/30) and *Leishmania (V.) shawi* 3.3% (1/30). As expected, the most common species was *L. (V.) guyanensis*. Surprisingly, none of the isolates were characterised as *L. (V.) brazi- liensis*. According to Romero et al. (2002b), cases of ATL caused by*L. (V.) braziliensis* are uncommon in nearby Manaus. Interestingly, the frequency of *L. (V.) naiffi* was 26.7% (8/30), which indicates that this species could be an etiologic agent of CL more frequent than expected in this region. These data corroborate those obtained by Figueira et al. (2008), who observed a relevant number of CL associated with*L. (V.) naiffi* in the municipalities of Rio Preto da Eva and Manaus.

Several methods have been used to define *Leishmania* species (Degrave et al. 1994, Romero et al. 2001a, Coelho et al. 2011). Nevertheless, MLEE remains one of the main tools for characterising*Leishmania* because it reveals polymorphisms that express phenotypes of population variations and taxonomically classify the different species of this parasite (Cupolillo et al. 1994). Precise identification of *Leishmania *spp is fundamental to understanding the disease’s epidemiology; improving the current knowledge concerning its pathology and the control measures (Coelho et al. 2011). Thus, it is likely that the transmission of *L. (V.) naiffi* in the Amazon Region is more frequent than has been reported. In this connection, the CL patients infected by*L. (V.) naiffi* (n = 8) are described below. All of them were men with a mean age of 37.4 ± 13.7 years (median = 37.5 years). The mean days of duration was 30 ± 24.8 days [median = 30 days (95% confidence interval: 22.5 - 52.5)]. The number of lesions ranged from one-four. Four subjects were initially treated with antimonial and four individuals were initially treated with pentamidine (antimonial: 10-20 mg Sb^+5^/kg/day for 20/30 consecutive days; pentamidine: 3 doses of 4 mg/kg with an interval of 72 h between doses) (Table II). Two of the patients experienced a poor response to antimonial or pentamidine therapy.

**TABLE II:** Demographic and clinical data of patients infected with*Leishmania (Viannia) naiffi*

Patient ID	Gender	Age	Lesions (n)	Location of lesion	Disease durations (days)	Presence of amastigotes	Treatment	Outcome
1	M	36	1	Upper limb	30	+	Antimonial (20 days) + antimonial (30 days)	Failure
2	M	39	1	Lower limb	90	+	Pentamidine (3 doses) + pentamidine (3 doses)	Failure
3	M	59	1	Upper limb	60	+	Antimonial (20 doses)	Cure
4	M	30	1	Upper limb	15	+	Antimonial (20 doses)	Cure
5	M	29	4	Upper limb	20	+	Pentamidine (3 doses)	Cure
6	M	39	2	Lower limb	30	+	Pentamidine (3 doses)	Cure
7	M	52	2	Face	30	+	Antimonial (20 doses)	Cure
8	M	15	1	Lower limb	30	+	Pentamidine (3 doses)	Cure

M: male; +: positive smears of scarifications.

Previous studies have suggested that the *Leishmania* species and the endemic area examined can be influenced by the cure rate (Romero et al. 2001b, Arevalo et al. 2007). In a recent study conducted in AM, the cure rates in *L. (V.) guyanensis *infection after treatment with antimonial or pentamidine were 55.5% and 58.1%, respectively (Neves et al. 2011). To date, all of the studies described in the literature have associated infection by *L. (V.) naiffi* with spontaneous healing or a good therapeutic response. However, the data obtained by our group showed therapeutic failure in a patient who was infected by *L. (V.) naiffi *and treated with pentamidine (R Vieira-Gonçalves, unpublished observations). Corroborating this finding, we herein illustrate two new CL cases that are associated with* L. (V.) naiffi *infection and presented pentamidine or antimonial-resistant lesions (Table II), which indicates that the clinical evolution of infection from this species may not be as favourable as described. No cases of spontaneously healing was seem probably because the short period of disease duration until the diagnosis establishment. The cases of *L. (V.) naiffi*reported herein underline that this species may not have a self-limiting nature, as previously described (Naiff et al. 1991, van der Snoek et al. 2009), and could not be as uncommon as referenced (Grimaldi Jr et al. 1991,Figueira et al. 2014). The present results highlight the importance of characterising *Leishmania* spp in areas that exhibit a sympatric circulation of *Leishmania* spp parasites to define the epidemiological importance of each of these species to human disease. This knowledge can improve the surveillance and therapeutic approaches used to achieve a clinical cure.
